# Denitrification rates in lake sediments of mountains affected by high atmospheric nitrogen deposition

**DOI:** 10.1038/s41598-020-59759-w

**Published:** 2020-02-20

**Authors:** Carlos Palacin-Lizarbe, Lluís Camarero, Sara Hallin, Christopher M. Jones, Jordi Catalan

**Affiliations:** 1 CREAF, Campus UAB, E08193 Cerdanyola del Vallès, Spain; 2 Center for Advanced Studies of Blanes, (CEAB–CSIC), Girona, Spain; 3 Swedish University of Agricultural Sciences, Department of Forest Mycology and Plant Pathology, Uppsala, Sweden; 4 Environmental Change Ecology Group (GECA), CSIC, Campus UAB, E08193 Cerdanyola del Vallès, Spain

**Keywords:** Element cycles, Climate-change mitigation, Microbial ecology, Atmospheric chemistry, Limnology

## Abstract

During the last decades, atmospheric nitrogen loading in mountain ranges of the Northern Hemisphere has increased substantially, resulting in high nitrate concentrations in many lakes. Yet, how increased nitrogen has affected denitrification, a key process for nitrogen removal, is poorly understood. We measured actual and potential (nitrate and carbon amended) denitrification rates in sediments of several lake types and habitats in the Pyrenees during the ice-free season. Actual denitrification rates ranged from 0 to 9 μmol N_2_O m^−2^ h^−1^ (mean, 1.5 ± 1.6 SD), whereas potential rates were about 10-times higher. The highest actual rates occurred in warmer sediments with more nitrate available in the overlying water. Consequently, littoral habitats showed, on average, 3-fold higher rates than the deep zone. The highest denitrification potentials were found in more productive lakes located at relatively low altitude and small catchments, with warmer sediments, high relative abundance of denitrification nitrite reductase genes, and sulphate-rich waters. We conclude that increased nitrogen deposition has resulted in elevated denitrification rates, but not sufficiently to compensate for the atmospheric nitrogen loading in most of the highly oligotrophic lakes. However, there is potential for high rates, especially in the more productive lakes and landscape features largely govern this.

## Introduction

Reactive nitrogen (N_r_) in the environment has at least doubled since preindustrial times due to human activities^1^. This anthropogenic alteration is one of the critical problems facing Earth-system processes^2^, as N_r_ can cause multiple effects across ecosystems until it is transformed back to nonreactive N_2_^3^ by denitrification^4,5^. This microbial process reduces nitrogenous oxides, mainly nitrate and nitrite, to dinitrogen gases N_2_O and N_2_ which are emitted to the atmosphere^6^. Freshwater ecosystems account for about 20% of global denitrification, and being hot spots for denitrification, they exceed the activity of soils per unit area on an annual basis^4^. Many mountain areas of the Northern Hemisphere have received large atmospheric loadings of N_r_ during the last decades^7–10^, resulting in elevated nitrate concentrations in mountain streams and lakes^7^. These waters are deficient in phosphorus (P), and therefore the supply of N usually exceeds the assimilation capacity by algae^7^. Thus, phytoplankton and benthic algae growth is P limited in mountain lakes affected by high N deposition^7,11^. Despite that N deposition can be homogeneous throughout a region^12^, nitrate accumulation in the lakes differs depending on internal and external P loads. In more productive lakes, the accumulation of organic C and N in the sediments is higher and nitrate remaining in the water column lower. Small lakes usually show higher productivity, particularly, if they are located at lower altitude as the growing season is longer and nutrient and organic matter (OM) loads from the surroundings increases^13,14^.

While nitrate accumulation in mountain lakes affected by N deposition is indicative of an altered N-cycle^15^, little is known about how the rates of the N-cycle pathways have been modified. In particular, information about denitrification rates in mountain lake sediments is rare despite its fundamental role as a sink of N_r_^16–20^. Sediments typically show higher denitrification rates than the water column^21^, but only a few studies have compared denitrification rates between deep and littoral lake zones^22–27^ and, in the latter, between vegetated and non-vegetated sediments^20,28–30^. Evaluating denitrification rates in the field is particularly challenging and may involve large uncertainties^15^. Denitrification dynamics can be episodic and spatially heterogeneous^31,32^. Such variation occurs not only due to fluctuations in resources (e.g., nitrate) and conditions (e.g., temperature) but also because denitrification is a facultative functional trait that is expressed in denitrifying microorganisms under micro-oxic or anoxic conditions. Furthermore, a large proportion of mountain lake sediments are found within the lake photic zone. Benthic algal communities and macrophytes influence denitrification processes by oxygen release, carbon (C) exudates, and N_r_ assimilation^20,29,30,33–37^. The specific macrophyte species also influence the redox profile of the sediment, for instance, isoetid and helophyte macrophytes oxygenate the sediment, while elodeids do not^38^. We have recently shown that the relative abundance of genes encoding enzymes catalysing different N-cycle processes differs among mountain habitats and lake characteristics^39^, with denitrification genes favoured in some of them. This suggests that the genetic potential for different N cycle process, and denitrification, in particular, varies in these systems. However, the relative degree to which the observed gene potentials and other ecological factors predict actual denitrification rates remains unknown.

Our aim was to investigate the relationship of actual, and substrate-induced denitrification rates, hereafter termed potential denitrification rates, with factors ranging from sediment to landscape features. Here, we considered genetic potentials, sediment conditions, water column physical and chemical variables, and holistic landscape descriptors. The ultimate objectives were to (i) identify factors explaining denitrification rates in mountain lake sediments and (ii) estimate if actual or potential denitrification rates can cope with the current atmospheric N load from deposition. Previous studies of denitrification activity in mountain lake sediments have used sediment slurries^17–20^. In contrast, we used a recently described protocol for measuring actual and potential denitrification rates in intact cores^40^. This approach avoids modification of substrate diffusion from the overlying water column, providing more realistic estimates of *in situ* and potential denitrification rates in the sediments.

## Methods

### Sites and sampling

The 11 lakes studied are situated in the central area of the Pyrenees within or nearby the Aigüestortes i Estany de Sant Maurici National Park (Table 1). The atmospheric N load from bulk atmospheric deposition in this area in 2010 was c. 10 kg N ha^−1^, matching the global average^10^. The lakes are dimictic, with a snow-ice cover during about half of the year, and ultra-oligotrophic (total phosphorus [TP] < 150 nM, except the oligotrophic Bassa de les Granotes where [TP] < 300 nM^41^) with circumneutral pH (~7)^42^.

**Table 1:** Description of the lakes studied sorted by decreasing nitrate concentrations in the water column.

Lake	Sediment habitat^a^	Latitude	Longitude	Altitude^b^	Lake area^b^	Catchment area^b^	Renewal time^b^	Depth^c^	TP^d^	NO_3_^−^
		(N)	(E)	(m a.s.l.)	(ha)	(ha)	(months)	(m)	(nM)	(μM)
Contraix	R, D	42.58874	0.91861	2572	9.3	100	9.9	59	49	15.5 ± 0.9
Bergús	D	42.58947	0.95717	2449	6.2	126	3.9	50	44	11.3 ± 0.6
Llebreta	C, R, D	42.55083	0.89031	1620	8	5438	0.1	12	89	10.6 ± 2.3
Redó Aigüestortes	D	42.58216	0.95949	2117	6.3	325	1.6	11	76	7.0 ± 1.0
Llong	R, D	42.57431	0.95063	2000	7.1	1111	0.6	13	89	6.3 ± 3.8
Gelat de Bergús	R, D	42.59106	0.96331	2493	1.4	24	2.3	8	42	5.0 ± 1.0
Redon	R, D	42.64208	0.77951	2235	24.1	153	36	73	58	4.8 ± 1.3
Pòdo	D	42.60307	0.93906	2450	4.6	33	9.4	25	75	1.0 ± 0.0
Bassa de les Granotes	D	42.57330	0.97124	2330	0.7	3	9.9	5	292	0.2 ± 0.4
Plan	E, I, C, D	42.62248	0.93070	2188	5	23	15.1	9	102	0.1 ± 0.4
Redon de Vilamòs	I	42.78078	0.76233	2209	0.6	12	1.7	5	NA	0

^a^Studied habitat: littoral sediments from rocky areas (R), helophyte (*Carex rostrata*) belts (C), beds of isoetid (I) and elodeid (E) macrophytes, and non-vegetated deep (D) sediments.

^b^Landscape descriptors used for modelling denitrification rates.

^c^Maximum water column depth.

^d^Total phosphorus^51^.

All main sediment habitats in the lakes were considered: vegetated littoral sediments with helophyte (C), elodeid (E) or isoetid (I) macrophytes, non-vegetated rocky littoral (R) and deep sediments (D). Some habitats were present in only a few lakes (Table 1). Plan lake is exceptionally rich in macrophytes, including the helophyte *Carex rostrata*, elodeids (*Potamogeton alpinus, P. berchtoldii and Myriophyllum alterniflorum*), and isoetids (*Isoetes palustris, I. setacea* and *Subularia aquatica*)^43^. During the ice-free period (June-November) of 2013 and 2014, a total of 146 sediment cores from 20 sites at 37 times were sampled. Sediments were collected using acrylic cores (ø 6.35 cm). Only undisturbed cores were used (i.e., >10 cm of sediment thickness with clear overlying water and sharp interface). Sediments around the deepest zone of the lake were sampled from an inflatable boat using a gravity corer^44^, while the littoral habitats were sampled manually by wading or snorkelling^40^.

### Denitrification rate measurements

The acetylene inhibition method, combined with sensors for nitrous oxide (N_2_O), was applied. This method inhibits the reduction of N_2_O to N_2_^45,46^ and enables reliable estimates of denitrification rates of at least 0.4–1 μmol N_2_O m^−2^ h^−1^ ^40^. This is the most used method to measure denitrification and has been used in all studies of denitrification in mountain lakes^16–20^, allowing comparisons across studies. However, a potential drawback is that incomplete inhibition of N_2_O reduction can occur or nitrification can be partially inhibited, which would underestimate the rates^47,48^. Measurements were performed in anoxic conditions during 12 h in an incubation chamber ensuring darkness and constant temperature (±1 °C) of 5, 10 or 15 °C, using the nearest temperature to that measured *in situ* at the time of sampling for each core. Anoxia and acetylene inhibition were achieved by first bubbling the overlaying water for 10 min N_2_ and then adding C_2_H_2_. The accumulated N_2_O in the water phase was measured using a modified Clark electrode probe (N_2_O-R microsensor, Unisense A/S, Denmark; detection limit ~0.1 μM). Gentle stirring was applied to prevent water stratification but avoiding sediment resuspension. Readings were taken every 5 min via a picoammeter logged to a laptop. The response of the electrochemical sensor is linear in the range of 0–1.2 mM^49^. The instrument was kept polarised during the measurement period and was calibrated at the measuring temperature using a calibration chamber, degassed deionised water and a freshly prepared ~20 μM N_2_O solution. The latter was obtained by adding a specific volume of N_2_O saturated water^50^ to the degassed water following manufacturer’s instructions. Further details about the method are provided in Palacin-Lizarbe, *et al*.^40^.

**Figure 1:**
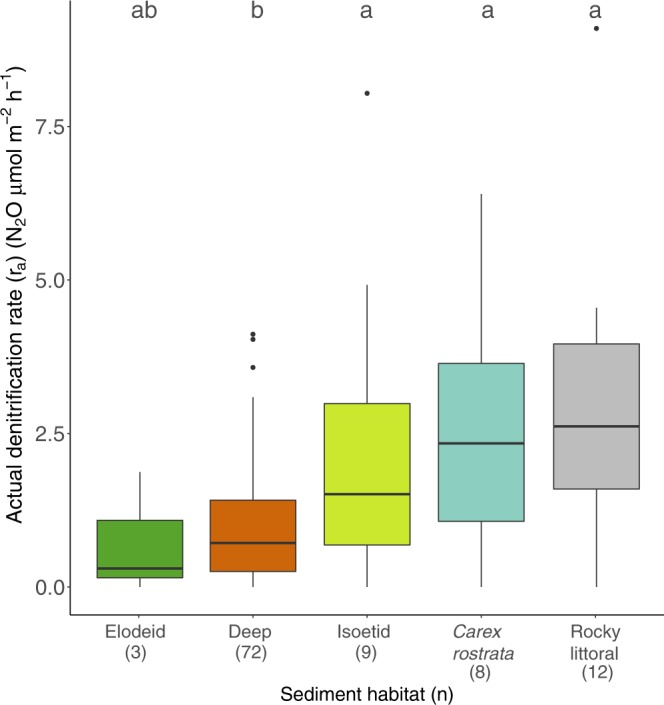
Actual denitrification rates (r_a_) in mountain lake sediments by habitat. Letters over each box indicate significant differences between habitats (Kruskal-Wallace test, p < 0.001, followed by pair-wise Mann-Whitney tests between habitats p < 0.05). Boxplots depict the interquartile range (box), median value (line), 1.5 x interquartile (whiskers), and outliers (points).

**Figure 2:**
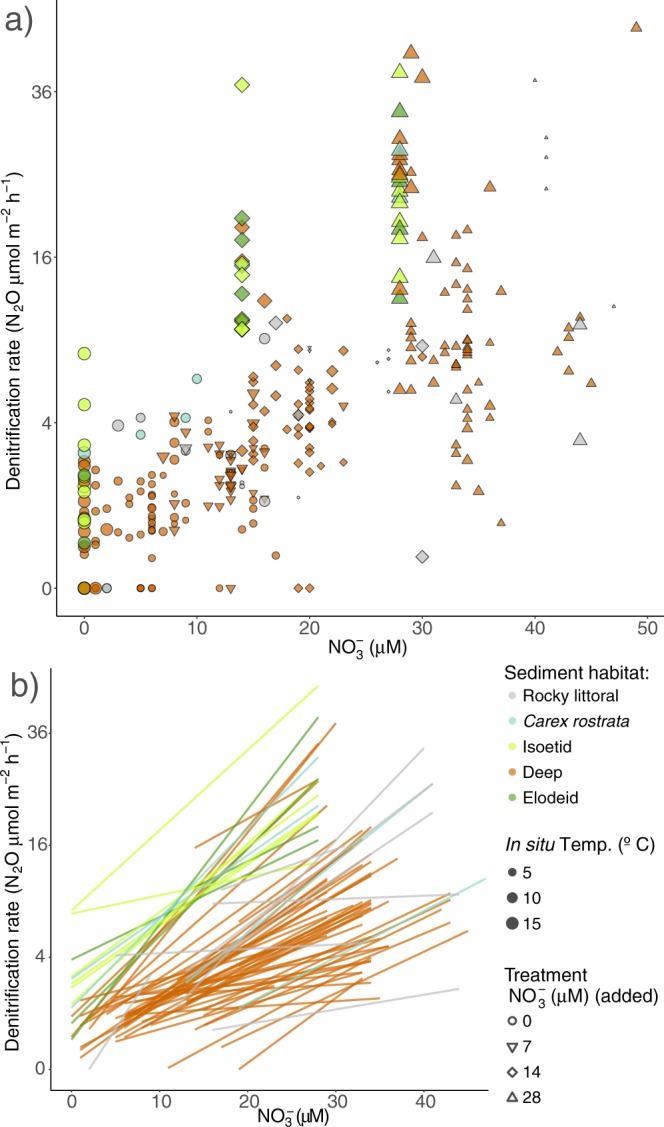
Denitrification rates in the sediments against the nitrate concentration in the water overlying the sediments (sum of initial and added nitrate). Note the square root scale in the y-axis. Colours indicate the habitats. In **(a)**, the symbol size is proportional to *in situ* temperature, and shape indicates the treatment (0, 7, 14 and 28 μM nitrate added, respectively). In **(b)**, lines are the linear models for each sediment core measured.

We performed 104 actual denitrification rates measured within less than ~4 h after the sediment core sampling without any substrate addition (Fig. 1), and 85 denitrification potential rates performed by adding nitrate (28 μM) and glucose (1.5 g/L) to the water phase of the core in which actual rates were previously measured. The actual rates should correspond to a lower bound of the range that can be found *in situ*, occurring when nitrate concentration is at the lowest seasonal values, usually summer stratification, and coupling with nitrification has little influence, whereas the potential rates will approach an upper bound in conditions of episodic high nitrate or high coupling with nitrification. The highest values historically measured in monitoring and surveys across lakes within the Pyrenean range did not show values above 28 μM nitrate^51,52^, thus this was the concentration selected for the additions. Occasionally, additions of 7 and 14 μM were also used to check for the continuity of response to nitrate between actual and potential measurements (Fig. 2). In a few cases (9), highly exaggerated nitrate concentrations (i.e. >300 μM) were added to evaluate upper rate limits. A total of 314 denitrification rates were estimated (Fig. 2).

### Water and sediment characterisation

Physical and chemical properties of the sediments, as well as the overlying water column, were characterised (Table S1). The temperature of the water overlying the sediment core was measured during sampling. For chemical analyses, water samples were filtered through a pre-combusted GF/F glass fibre filter and analysed as recently reported^39^. Briefly, nitrate and sulphate were determined by capillary electrophoresis, while ammonium and nitrite were determined by colourimetric methods. Dissolved organic carbon was measured by catalytic combustion to CO_2_ and detection by IR spectroscopy.

The upper sediment layer (0–0.5 cm depth) always showed the highest actual and potential denitrification activities in preliminary experiments with slurries of sliced sediment cores, in accordance with previous studies of non-vegetated marine and estuarine sediments^53,54^. Therefore, only sediment descriptors from this layer were considered in this study. Around 5 mg of the freeze-dried sediment was encapsulated together with a catalyst (Va_2_O_5_) in tin capsules for the determination of C and N content and isotopic composition, performed at the University of California Davis Stable Isotope Facility. The dry weight percentage of OM content in the samples was determined using the loss on ignition (LOI) procedure following Heiri, *et al*.^55^. The sediment density was determined using a pycnometer and rehydrating a known amount of freeze-dried sediment. The median grain size of the sediment was determined by laser diffraction (Mastersizer 2000, Malvern Instruments Ltd, UK). Freeze-dried sediment was rehydrated in distilled water and introduced into the sample dispersion unit (Hydro 2000 G, Malvern Instruments Ltd, UK) adding hexametaphosphate and sonicating to avoid aggregates. Laser obscuration was between 10–20% and the measuring range between 0.02 and 2000 μm.

Sediment molecular descriptors used in this study (i.e. DNA content, abundance of 16 S rRNA gene, and functional genes involved in denitrification: *nirS*, *nirK*, *nosZI*, and *nosZII*; Table S1) are a subset of data previously published in Palacin-Lizarbe, *et al*.^39^.

### Statistical methods

Multiple linear regression models were developed to investigate the degree to which different factors explain both actual and potential denitrification rates. First, models were initially fit for each category of factor variables (i.e. molecular, sediment, water, and landscape). Then, an overall model was built based on the variables selected in each category. In this way, the explanatory capacity of the environment relative to genetic potentials could be investigated at different spatial scales^56^. Tables 1 and S1 list all measured descriptors included in the modelling. All calculations were performed using R version 3.4.3^57^.

All variables were standardised to z-scores to obtain regression coefficients that are proportional to the influence of each explanatory variable in the different models (Tables 2 and S3), such that their relative importance can be immediately evaluated. Before being scaled, some variables were square root or log_10_ transformed to reduce the influence of extreme values. We developed alternative mixed regression models for each category of explanatory variables that include lake and habitat as random factors to account for the unquantified lake- or habitat-specific variation. We further tested habitat influence by including its presence/absence in the general models (e.g., model 5c) to consider habitat features not accounted by the measured descriptors. To avoid overfitting, we selected the explanatory variables included in the models using the *dredge* function of *MuMIn* package^58^, and models in which all variables were significant at p < 0.05 were selected. Because not all descriptors were available for each sample, the number of final actual denitrification rates estimations was reduced from the initial 104 in the complete dataset (Fig. 1) to 69 in the models (Table 2). Regression models were fitted with the *lm* function of the R core package stats^57^, and mixed models with the *lme* function of the package *nlme*^59^. Best fitting models were selected based on Akaike’s information criterion for small sample size (AICc) and R^2^ determined by the *anova* function of the package *stats*^57^. When comparing mixed and fixed-effects models using *anova*, the latter were fitted with the *gls* function of the package *nlme*^59^. All models selected showed p < 0.001. Kruskal-Wallis (KW) and Mann-Whitney U-tests were used for sample set comparisons.

**Table 2:** Multiple linear regression models relating the actual (r_a_, models 1–5) and potential (r_p_, 28 μM nitrate added, models 6–12) denitrification rates to several types of explanatory variables. The models presented had the highest explanatory power compared to other models within each category of predictor variables (Table S3).

Model	Explanatory variables	Formula: Fixed//random	AICc	Fixed R2.(Global R2)
		Actual denitrification rates (n = 69)		
1a	Sediment (molecular)	$\sqrt{{r}_{a}}=0.62\times {\rm{log10}}(nosZII)-0.48\times \sqrt{nirS}-0.32\times \sqrt{{\rm{DNA}}}$	192	0.18
2a	Sediment (abiotic)	$\sqrt{{r}_{a}}=-1.16\times {\rm{N}}+0.85\times {\rm{C}}$	188	0.20
3a	Water (abiotic)	$\sqrt{{r}_{a}}=0.61\times {{{\rm{NO}}}_{3}}^{-}+0.46\times {\rm{Temperature}}$	185	0.24
3c	Water (abiotic)	$$\begin{array}{rcl}\sqrt{{r}_{a}} & = & 0.09+0.90{{\times {\rm{NO}}}_{3}}^{-}+0.59\times {\rm{Temperature}}\\ & & //{\rm{random}}= \sim \,1+{{{\rm{NO}}}_{3}}^{-}|{\rm{lake}}\end{array}$$	193	0.38 (0.48)
4	Landscape	$\sqrt{{r}_{a}}=0.30{\times \log }_{10}({\rm{Catchment}})$	195	0.08
**5a**	**All**	$$\begin{array}{rcl}\sqrt{{{\boldsymbol{r}}}_{{\boldsymbol{a}}}} & {\boldsymbol{=}} & {\bf{0.58}}{\boldsymbol{\times }}{\boldsymbol{Temperature}}{\boldsymbol{+}}{\bf{0.37}}{\boldsymbol{\times }}{\boldsymbol{N}}{{\boldsymbol{O}}}_{{\bf{3}}}^{{\boldsymbol{-}}}{\boldsymbol{-}}{\bf{0.29}}{\boldsymbol{\times }}{\boldsymbol{N}}{\boldsymbol{+}}{\bf{0.26}}{\boldsymbol{\times }}\,{\bf{\log }}\,{\bf{10}}{\boldsymbol{(}}{\boldsymbol{Catchment}}{\boldsymbol{)}}\\ & & {\boldsymbol{-}}{\bf{0.24}}{\boldsymbol{\times }}\sqrt{{\boldsymbol{DNA}}}\end{array}$$	**177**	**0.38**
5c	All	$$\begin{array}{rcl}\sqrt{{r}_{a}} & = & 0.10+0.93\times {{{\rm{NO}}}_{3}}^{-}+0.71\times {\rm{Temperature}}-0.27\times \sqrt{nirS}\\ & & //{\rm{random}}= \sim \,1+{{{\rm{NO}}}_{3}}^{-}|{\rm{lake}}\end{array}$$	193	0.44 (0.54)
		Potential denitrification rates (n = 52)		
6c	Sediment (molecular)	$$\begin{array}{rcl}\sqrt{{r}_{p}} & = & 0.42+0.68\times \sqrt{nirS}-0.65\times {\rm{log10}}\,(nosZI)\\ & & //{\rm{random}}= \sim 1|{\rm{habitat}}\end{array}$$	136	0.35 (0.55)
7a	Sediment (abiotic)	$\sqrt{{r}_{p}}=0.49\times {\rm{C}}+0.43{\times {\rm{\delta }}}^{15}{\rm{N}}$	124	0.46
8	Water (abiotic)	$\sqrt{{r}_{p}}=0.48\times {\rm{Temperature}}+0.48{\times \log }_{10}({{{\rm{SO}}}_{4}}^{-2})-0.43{{\times {\rm{NO}}}_{3}}^{-}$	115	0.53
9a	Landscape	$\sqrt{{r}_{p}}=0.75\times {\rm{Altitude}}-0.65{\times \log }_{10}({\rm{Catchment}})$	117	0.52
**10a**	**All**	$$\begin{array}{rcl}\sqrt{{{\boldsymbol{r}}}_{{\bf{p}}}} & {\boldsymbol{=}} & {\boldsymbol{-}}{\bf{0.77}}{\boldsymbol{\times }}{\bf{l}}{\bf{o}}{\bf{g}}{\bf{10}}{\boldsymbol{(}}{\boldsymbol{nosZI}}{\boldsymbol{)}}{\boldsymbol{+}}{\bf{0.69}}{\boldsymbol{\times }}{\bf{T}}{\bf{e}}{\bf{m}}{\bf{p}}{\bf{e}}{\bf{r}}{\bf{a}}{\bf{t}}{\bf{u}}{\bf{r}}{\bf{e}}{\boldsymbol{+}}{\bf{0.64}}{\boldsymbol{\times }}\sqrt{{\boldsymbol{nirS}}}{\boldsymbol{-}}{\bf{0.47}}{\boldsymbol{\times }}{\bf{N}}\\ & & {\boldsymbol{+}}\,{\bf{0.34}}{\boldsymbol{\times }}{\bf{l}}{\bf{o}}{\bf{g}}{\bf{10}}{\boldsymbol{(}}{\bf{S}}{{\bf{O}}}_{{\bf{4}}}^{{\boldsymbol{-}}{\bf{2}}}{\boldsymbol{)}}\,{\boldsymbol{-}}{\bf{0.33}}{\boldsymbol{\times }}{\bf{A}}{\bf{l}}{\bf{t}}{\bf{i}}{\bf{t}}{\bf{u}}{\bf{d}}{\bf{e}}\end{array}$$	**90**	**0.77**

Note: The intercept of all models was always not significant (p > 0.05), it was not shown when has negligible value (<1 × 10^−15^). In bold are indicated the best models, i.e. trade-off between being simple and explicative (AICc).

## Results

### Actual denitrification rates and potential denitrification rates

Actual denitrification rates (r_a_) ranged from 0 to 9 μmol N_2_O m^−2^ h^−1^ (Fig. 1), with a mean of 1.5 ± 1.6 μmol N_2_O m^−2^ h^−1^ (mean ± SD). The rates differed significantly among habitats (KW p < 0.001, Table S2), with sediments from all littoral habitats except elodeids exhibiting r_a_ values that averaged 2.8-fold higher than those in the deep zone (Fig. 1, MW p < 0.05). Rates in the sediments with isoetids were significantly correlated with the density of plants (Pearson’s R = 0.83 p < 0.01).

The potential rates (r_p_, 28 μM nitrate added) also differed among habitats, but ranked differently than the actual rates (C ≈ I ≈ E > R > D, KW p < 0.001, Table S2) and r_p_ and r_a_ were not correlated (Pearson’s R = 0.20, p = 0.13). Using realistic nitrate additions of 7, 14, and 28 μM, increasing denitrification rates with nitrate added was observed (Fig. 2). The denitrification rates measured after addition of a high nitrate concentration (i.e. >300 μM) were higher in the corresponding habitat and lake and ranged from 11 to 186 μmol N_2_O m^−2^ h^−1^. Overall, these observations indicate that the capacity for denitrification in the lakes is never nitrate saturated under natural conditions, except for a few non-vegetated sediments from the rocky littoral and from the deep zone (Fig. 2).

### Factors explaining actual denitrification rates

Models of actual rates in response to sediment, water and landscape factors, as well as all predictors combined (models 1–5; Table 2) explained up to 44% of the variation observed in r_a_. Nitrate concentration and temperature provided the most explanatory power (Table 2 and Table S3). These descriptors were always part of the water category (model 3) and the general models. Nitrate concentration showed higher positive influence in r_a_ than temperature, especially in the models including the lake or habitat as a random effect (models 3c and 5c, Table 2). The best general model (5a) also included the catchment area and sediment DNA and N content.

Models examining only molecular predictor variables included *nosZI and nosZII* gene abundances as being positively associated with r_a_, whereas *nirS* gene abundances and DNA content had negative coefficients (1a, Table 2; 1b, Table S3). However, explicative capacity was lower than that observed for water-associated variables (≤18%). Similarly, the abiotic sediment factors were poor descriptors. The absolute content of N and C were more explicative than the stoichiometric ratio (C/N) ratio, which was included in the modelling but not selected. More specifically, N content showed a greater negative effect than the positive effect of C (model 2a, Table 2). The landscape models showed the lowest explicative power among categories (model 4, Table 2).

### Factors explaining potential denitrification rates

The models explaining potential rates (models 6–12, Table 2) performed better than those developed for actual rates: up to 79% of the r_p_ variation by the general models, and always more than 45% for any of the category models. General models without molecular or landscape factors still explained about 65% of the variation (models 11 and 12, respectively, Table S3). Similar to models for r_a_, the best general model for predicting r_p_ (10a, Table 2) included factors from all categories, including *nosZI* and *nirS* gene abundances, N content, water temperature, sulphate concentration, and lake altitude. Gene abundances, temperature, and altitude were also selected in models including the presence/absence of isoetids and *Carex* or the lake effect (10b and 10c, Table S3). No model that included the presence/absence of any habitat, or lake or habitat as a random factor was significantly better than the best fixed-effect general models 5a and 10a for r_a_ and r_p_, respectively. Therefore, the included factors captured any lake- or habitat-specific variation.

Abundances of *NosZI* and *nirS* gene were the selected factors in the molecular model category, with negative and positive influences on r_p_, respectively (model 6c, Table 2). These genes and temperature were factors with higher coefficients in the general models of r_p_. Regarding abiotic sediment factors, δ^15^N signature was always selected with either C, N, or OM content as accompanying variable, all with positive influence (models 7a-c, Tables 2 and S3). In contrast, when N content was selected in the best general model (10a, Table 2), it showed a negative influence. Interestingly, nitrate concentration was negatively associated with r_p_ in the best model of water variables only, whereas temperature and sulphate concentrations had a positive influence on r_p_ (model 8, Table 2). Both altitude and catchment area were significant factors in the landscape model (9a, Table 2), with negative influences on r_p_. The role of landscape factors showed a contrasting influence on r_a_ and r_p_, very low in the former (<10%) and high in the latter (~50%).

## Discussion

Our results indicate that denitrification in mountain lake sediments is mostly nitrate limited. The rates estimated in this study show values within the range of other mountain lakes^18,19^, although slightly lower if similar nitrate concentration and temperature are considered. This difference could potentially be explained by differences in efficiency of the acetylene inhibition, with incomplete inhibition resulting in underestimations of denitrification rates^47,48^. The other studies have been done in sediment slurries, whereas our study was done with intact cores that may have less efficient inhibition due to slow diffusion of acetylene into the sediments. However, whole-core incubations have the advantage of creating more realistic conditions for estimates of *in situ* rates.

Nitrate levels were more important in predicting actual rates compared to temperature, which has been observed in other studies^16,60^. In littoral habitats, higher nitrate availability through enhanced diffusion by wave action and inputs via runoff and groundwater flows, in combination with warmer temperatures give rise to conditions that are more favourable to denitrification, which can explain the higher actual rates. Similarly, high littoral denitrification rates were found in an oligo/mesotrophic lake (Gull Lake, Michigan, USA)^22^. The more permanent aerobic conditions in the shallower littoral habitats may result in better coupling between nitrification and denitrification, as suggested by previous results showing higher abundance of ammonia-oxidizing archaea and denitrifiers in littoral habitats^39^. In natural settings, darkness and anoxia prevail in the sediments during many hours of the day (e.g., night) and year (e.g. ice-covered period). These conditions are therefore appropriate for comparing the range of activity between different zones of the lake. However, incubating under anoxic conditions does not consider coupled nitrification-denitrification and, also, the acetylene inhibition method partially blocks nitrification and thereby may underestimate *in situ* activities^48^. Thus, for measuring coupled nitrification-denitrification rates, other incubation conditions and another method to determine denitrification activity is needed. Overall, this suggests that the rates could be underestimated in the shallower littoral habitats. Habitats with a more reductant sediment profile, such as sediments with elodeid macrophytes and from the deep part of the lake^38^, exhibited lower actual rates. Although the differences in rates among habitats were according to expectations, the importance of the lake zone may also change depending on the season, something demonstrated in a eutrophic boreal lake^23^.

The potential denitrification rates were positively related to mountain lake productivity, which suggests a dependency on landscape features. Altitude determines many variables associated with overall lake productivity (e.g. nutrient availability, temperature, and growth period duration), whereas catchment area affects more the quality of OM by modifying the relative contribution of autochthonous vs allochthonous sources. Generally, OM produced within the lake is more labile and has a higher quality (e.g. lower C/N); thus small catchments favour autochthonous contributions. The negative relationship observed between nitrate levels and potential rates is likely due to nitrate depletion in the more productive lakes during the ice-free period, which has been observed previously in sediments of the Laurentian great lakes^61^. In the Rocky mountains, potential denitrification rates correlated with the sediment P/C ratio, a surrogate of productivity^19^. In fact, denitrifier distribution is related to productivity. In a recent study, we showed that *nirS*-types dominated in the more productive sediments, while *nosZI-*type displayed higher relative abundances in more oligotrophic sediments (e.g. rocky littoral sediments of alpine lakes)^39^. These relationships support the opposed role of *nirS* and *nosZI* gene pools in explaining actual and potential denitrification rates in the present study. The role of sulphate for potential denitrification rates was less expected. Some bacteria can shift between O_2_, NO_3_^−^/NO_2_^−^, and SO_x_ respiration, using the latter when the other electron acceptors with a higher energy benefit are depleted^62^. Mountain lake sediments are environments of contrasting seasonal conditions that may result in fluctuations of the different electron acceptors, which will favour these facultative bacteria. If this could explain the positive influence of sulphates in the potential denitrification rates need to be further explored and verified.

The increase in the N_r_ deposition should have enhanced denitrification rates in mountain lakes provided that nitrate was limiting the actual rates. However, as the denitrification potential related more to productivity than to nitrate availability, the enhancement is likely not sufficient to compensate the higher loading due to N_r_ deposition, resulting in nitrate accumulation in the water column of the most oligotrophic sites. Nevertheless, the denitrification potential was not saturated, indicating a high capacity for denitrification that could be realized if conditions change. Thus, the lake bacteria could theoretically mediate high N_r_ deposition, particularly in the more productive lakes. Current atmospheric nitrogen deposition in the area is about 10 kg N ha^−1^ y^−1^, which corresponds to the rates in between the actual and potential denitrification rates; on average, 2 and 18 kg N ha^−1^ y^−1^, respectively. We cannot calculate a net balance of N_r_ in the studied lakes, as they also receive an unknown amount of the N_r_ deposited in the catchment, and the contribution of N_2_-fixation is unknown. Nevertheless, if we only consider the nitrate concentrations found in the lakes and the actual rates measured, a simple calculation indicates that the N_r_ removal from lakes by denitrification would require about 1060 days according to the actual rates, assuming that nitrate reduction did not decelerate during nitrate depletion. With a removal time longer than one year, nitrate accumulates. However, when basing the calculation on potential rates, it would take less than a year (139 days) for removal, which will deplete the nitrogen in the lakes. The estimated potential rates in the more productive lakes, where nitrate is depleted in summer, indicate that denitrification might have a significant role as a sink of the enhanced N deposition. However, nitrate remains high in the water column in most of the oligotrophic lakes, showing that denitrification cannot cope with the enhanced N deposition in their watersheds. Our estimates are based on the ice-free period, where actual denitrification rates were higher in the more oligotrophic sediments with higher nitrate availability and lower sediment DNA and N content than in more productive lakes. However, the water column in the productive lakes was nitrogen depleted, likely due to the uptake by primary producers. During winter, the conditions will change, and nitrate availability will be higher in these lakes^52^ as there is almost no competition with primary producers. Therefore, *in situ* rates could approach the potential rates, especially in the more productive lakes during the winter period. Denitrification activity in winter and under ice deserves further investigation to understand the whole year effect of N_r_ on denitrification in mountain lakes.

## Supplementary information

Supplementary Information.

## Data Availability

The datasets generated during the current study, i.e. the denitrification rates dataset, and the landscape, water, and sediment factors dataset, are deposited to Dryad. Accession No. is 10.5061/dryad.j6q573n95.
