# Supplementary material for: Denitrification rates in lake sediments of mountains affected by high atmospheric nitrogen deposition
Source: Sci Rep. 2020 Feb 20;10:3003. doi: 10.1038/s41598-020-59759-w (PMC7033281; doi:10.1038/s41598-020-59759-w)
Supplement: Supplementary file 1 — Supplementary Information. [file 41598_2020_59759_MOESM1_ESM.pdf]

SUPPLEMENTARY INFORMATION for

Denitrification rates in lake sediments of mountains affected by high atmospheric nitrogen deposition

Carlos Palacin-Lizarbe[1], Lluís Camarero[2], Sara Hallin[3], Christopher M Jones[3], and Jordi Catalan[1, 4]

[1]CREAF, Campus UAB, Cerdanyola del Vallès, Spain, [2]Center for Advanced Studies of Blanes, (CEAB–CSIC), Girona, Spain, [3]Swedish University of Agricultural Sciences, Department of Forest Mycology and Plant Pathology, Uppsala, Sweden, [4]CSIC, Cerdanyola del Vallès, Spain

Current file content:

- **Table S1**. Sediment and water descriptors by habitat.
- **Table S2**. Actual (r_a, no nitrate added) and potential (r_p, 28 μM nitrate added) denitrification rates in mountain lake sediments.
- **Table S3**. Multiple linear regression models relating the actual and potential denitrification rates to several types of explanatory variables.

Table S1. Sediment and water descriptors by habitat.

			Sediment habitat [a]					
			All	R	C	I	D	E
Temperature [b]	° C	**mean**	**9.1**	**8.4**	**9.8**	**16.5**	**7.4**	**16.4**
		SD	5.3	4.3	6.5	0.8	4.5	0.4
		min	0.8	0.8	0.8	15.0	3.6	16.2
		max	17.5	12.9	16.2	17.5	16.1	16.9
$[NO_3^-]$ [b]	µM	**mean**	**5**	**9**	**6**	**0**	**5**	**0**
		SD	6	7	6	0	6	0
		min	0	2	0	0	0	0
		max	17	16	13	0	17	0
$[NO_2^-]$ [b]	µM	**mean**	**0.10**	**0.15**	**0.09**	**0.06**	**0.10**	**0.04**
		SD	0.06	0.07	0.04	0.04	0.06	0.02
		min	0.02	0.07	0.04	0.03	0.03	0.02
		max	0.27	0.21	0.15	0.14	0.27	0.06
$[NH_4^+]$ [b]	µM	**mean**	**5.6**	**5.6**	**8.8**	**1.0**	**6.3**	**1.0**
		SD	9.4	9.5	9.9	0.1	10.2	0.2
		min	0.8	1.0	1.2	0.8	1.0	0.8
		max	51.3	25.0	25.0	1.2	51.3	1.2
$[SO_4^{2-}]$ [b]	µM	**mean**	**14**	**18**	**24**	**10**	**14**	**11**
		SD	7	10	15	1	5	0
		min	6	6	7	9	6	10
		max	40	32	40	13	27	11
DOC [b]	$mg * L^{-1}$	**mean**	**66**	**52**	**119**	**94**	**58**	**57**
		SD	53	32	108	33	48	5
		min	2	8	2	60	6	52
		max	301	86	233	155	301	62
z (water column depth) [b, c]	m	**mean**	**17.4**	**0.5**	**0.5**	**0.5**	**25.3**	**0.5**
		SD	22.8	0.0	0.0	0.0	23.9	0.0
		min	0.5	0.5	0.5	0.5	4.0	0.5
		max	72.0	0.5	0.5	0.5	72.0	0.5
Organic matter [d]	%	**mean**	**32**	**6**	**29**	**50**	**32**	**49**
		SD	14	5	18	12	9	6
		min	1	1	6	30	17	42
		max	66	14	48	66	48	54
Nitrogen (N) [d]	%	**mean**	**1.39**	**0.20**	**1.10**	**1.82**	**1.46**	**1.99**
		SD	0.59	0.21	0.81	0.41	0.42	0.30
		min	0.02	0.02	0.08	1.21	0.76	1.75
		max	2.44	0.57	1.93	2.44	2.34	2.32
$\delta^{15}N$ [d]	‰	**mean**	**-0.92**	**1.37**	**-0.32**	**0.02**	**-1.46**	**-0.68**
		SD	1.73	1.75	0.71	0.89	1.65	0.81
		min	-4.36	-1.10	-1.09	-1.14	-4.36	-1.52
		max	3.52	3.52	0.45	1.42	1.93	0.09
Carbon (C) [d]	%	**mean**	**14.24**	**2.73**	**13.54**	**21.83**	**14.09**	**20.51**
		SD	6.48	2.54	8.99	5.45	4.42	2.26
		min	0.20	0.20	2.01	14.05	7.47	18.43
		max	29.35	6.02	22.96	29.35	24.18	22.91
$\delta^{13}C$ [d]	‰	**mean**	**-24.45**	**-23.52**	**-23.44**	**-20.49**	**-25.70**	**-19.02**
		SD	3.53	2.59	3.82	2.68	2.96	1.07
		min	-31.73	-27.54	-26.61	-24.36	-31.73	-20.10
		max	-17.47	-20.11	-18.62	-17.47	-21.26	-17.96
C/N [d]	a/a	**mean**	**12.49**	**16.06**	**17.53**	**14.08**	**11.25**	**12.09**
		SD	3.10	4.32	6.75	2.30	0.99	0.50
		min	9.38	12.35	13.52	11.64	9.38	11.52
		max	29.52	22.82	29.52	18.17	15.21	12.45
Dry weight / wet weight [d]		**mean**	**0.13**	**0.56**	**0.23**	**0.08**	**0.08**	**0.08**
		SD	0.17	0.29	0.24	0.04	0.03	0.01
		min	0.02	0.25	0.09	0.05	0.02	0.08
		max	0.88	0.88	0.65	0.18	0.14	0.09
Sediment density [d]	$g * cm^{-3}$	**mean**	**1.55**	**2.21**	**1.72**	**0.83**	**1.60**	**0.99**
		SD	0.47	0.45	0.48	0.37	0.29	0.24
		min	0.45	1.59	1.29	0.45	1.10	0.84
		max	2.64	2.64	2.48	1.41	2.49	1.27
Sediment grain size [d]	µm	**mean**	**328**	**438**	**282**	**361**	**308**	**394**
		SD	90	130	135	107	63	58
		min	130	288	130	182	197	328
		max	627	627	433	511	440	435

			Sediment habitat [a]					
			All	R	C	I	D	E
[DNA] [e]	ng * m^{-2}	**mean**	**1.6E+08**	**2.5E+08**	**1.3E+08**	**9.5E+07**	**1.6E+08**	**1.2E+08**
		SD	9.8E+07	2.3E+08	5.3E+07	5.2E+07	7.6E+07	1.8E+07
		min	9.9E+05	4.3E+07	6.8E+07	5.1E+07	9.9E+05	1.0E+08
		max	7.0E+08	7.0E+08	2.0E+08	1.8E+08	3.3E+08	1.4E+08
[16S] [e]	copies * m^{-2}	**mean**	**4.5E+13**	**5.9E+13**	**4.2E+13**	**2.2E+13**	**4.9E+13**	**3.0E+13**
		SD	3.5E+13	3.8E+13	1.2E+13	1.1E+13	3.9E+13	2.8E+12
		min	3.2E+11	1.3E+13	2.6E+13	1.0E+13	3.2E+11	2.6E+13
		max	2.1E+14	1.1E+14	5.3E+13	3.8E+13	2.1E+14	3.1E+13
[*nirS*] [e]	copies * m^{-2}	**mean**	**3.3E+12**	**1.0E+12**	**1.7E+12**	**3.1E+12**	**3.8E+12**	**4.5E+12**
		SD	3.2E+12	6.4E+11	1.7E+12	3.5E+12	3.5E+12	6.0E+11
		min	1.6E+10	2.7E+11	4.4E+11	6.8E+11	1.6E+10	4.0E+12
		max	1.4E+13	1.8E+12	4.7E+12	1.1E+13	1.4E+13	5.1E+12
[*nirK*] [e]	copies * m^{-2}	**mean**	**1.4E+11**	**3.7E+11**	**1.4E+11**	**6.4E+10**	**1.2E+11**	**9.1E+10**
		SD	1.8E+11	4.2E+11	7.3E+10	3.9E+10	1.4E+11	1.4E+10
		min	7.8E+08	5.9E+10	5.1E+10	3.2E+10	7.8E+08	7.7E+10
		max	1.2E+12	1.2E+12	2.5E+11	1.5E+11	8.8E+11	1.1E+11
[*nosZ1*] [e]	copies * m^{-2}	**mean**	**1.2E+11**	**4.2E+11**	**6.6E+10**	**5.7E+10**	**9.5E+10**	**5.2E+10**
		SD	1.9E+11	5.6E+11	3.9E+10	4.9E+10	8.8E+10	1.5E+10
		min	4.4E+08	4.6E+10	3.1E+10	1.3E+10	4.4E+08	4.2E+10
		max	1.5E+12	1.5E+12	1.3E+11	1.4E+11	4.2E+11	6.9E+10
[*nosZ2*] [e]	copies * m^{-2}	**mean**	**4.5E+10**	**1.1E+11**	**1.8E+10**	**3.0E+10**	**4.3E+10**	**3.1E+10**
		SD	6.5E+10	1.8E+11	1.6E+10	2.8E+10	4.1E+10	1.3E+10
		min	1.8E+08	4.8E+09	1.0E+10	3.6E+09	1.8E+08	1.8E+10
		max	4.8E+11	4.8E+11	4.6E+10	7.7E+10	2.0E+11	4.4E+10

[a] Habitats: littoral sediments from rocky areas (R), helophyte (*Carex rostrata*) belts (C), beds of isoetids (I) and elodeid (E) macrophytes, and non-vegetated deep (D) sediments.

[b] Water (overlaying the sediment core) and sediment ([d] abiotic or [e] molecular) descriptors used for modelling denitrification rates. Sediment molecular data used in this study is a subset of data previously published in Palacin-Lizarbe, et al. (2019) *Front Microbiol*. DNA and gene abundances expressed in ng DNA m^{-2} or gen copies m^{-2}.

[c] Water column depth coded as 0.5 m for littoral sediments.

[f] Dry weight (DW).

Table S2. Actual (r_a, no nitrate added) and potential (r_p, 28 μM nitrate added) denitrification rates in mountain lake sediments.

		Sediment habitat [a]					
		**All**	**R**	**C**	**I**	**D**	**E**
**Actual denitrification rates (r_a) (μmol N_2O m^{-2} h^{-1})**	**mean**	**1.5**	**3.0**	**2.6**	**2.4**	**1.0**	**0.7**
	SD	1.6	2.3	2.1	2.6	1.0	1.0
	min	0.0	0.0	0.0	0.0	0.0	0.0
	max	9.1	9.1	6.4	8.0	4.1	1.9
**Potential denitrification rates (r_p) (μmol N_2O m^{-2} h^{-1})**	**mean**	**14.6**	**17.9**	**22.2**	**22.8**	**12.3**	**22.1**
	SD	10.2	12.9	7.5	7.9	9.6	8.8
	min	1.4	3.2	11.6	14.1	1.4	12.3
	max	45.8	37.6	28.0	38.8	45.8	33.1

[a] Studied sediment habitats sorted by the mean r_a: littoral sediments from rocky areas (R), helophyte (*Carex rostrata*) belts (C), beds of isoetids (I) and elodeid (E) macrophytes, and non-vegetated deep (D) sediments.

Table S3. Multiple linear regression models relating the actual (r_a, models 1-5) and potential (r_p, 28μM nitrate added, models 6-12) denitrification rates to several types of explanatory variables.

Model	Explanatory var	Formula: Fixed part	Random part	AICc (gls)	Fixed R^2	Global R^2
		Actual denitrification rates (n = 69)				
lake		$\sqrt{r_a} = 0.05$	~1|lake	202	0	0.24
habitat		$\sqrt{r_a} = 0.31$	~1|habitat	195	0	0.22
1a	Sediment (molecular)	$\sqrt{r_a} = 0.62\times\log_{10}(nosZII) - 0.48\times\sqrt{nirS} - 0.32\times\sqrt{\mathrm{DNA}}$	—	192 (201)	0.18	0.18
1b	Sediment (molecular)	$\sqrt{r_a} = 0.49\times\log_{10}(nosZI) - 0.32\times\sqrt{\mathrm{DNA}} - 0.29\times\sqrt{nirS}$	—	192 (202)	0.17	0.17
2a	Sediment (abiotic)	$\sqrt{r_a} = -1.16\times\mathrm{N} + 0.85\times\mathrm{C}$	—	188 (193)	0.20	0.20
2b	Sediment (abiotic)	$\sqrt{r_a} = 0.25 - 0.35\times\mathrm{N}$	~1|habitat	193	0.11	0.28
2c	Sediment (abiotic)	$\sqrt{r_a} = 0.32 - 0.33\times\mathrm{OM}$	~1|habitat	194	0.10	0.28
2d	Sediment (abiotic)	$\sqrt{r_a} = 0.31 - 0.30\times\mathrm{C}$	~1|habitat	195	0.08	0.27
3a	Water (abiotic)	$\sqrt{r_a} = 0.61\times NO_3^- + 0.46\times\mathrm{Temperature}$	—	185 (192)	0.24	0.24
3b	Water (abiotic)	$\sqrt{r_a} = 0.06 + 0.71\times NO_3^- + 0.57\times\mathrm{Temperature}$	~1|lake	193	0.28	0.40
3c	Water (abiotic)	$\sqrt{r_a} = 0.09 + 0.90\times NO_3^- + 0.59\times\mathrm{Temperature}$	~1+NO_3^-|lake	193	0.38	0.48
3d	Water (abiotic)	$\sqrt{r_a} = 0.34 + 0.37\times NO_3^-$	~1|habitat	189	0.12	0.33
3e	Water (abiotic)	$\sqrt{r_a} = 0.23 + 0.53\times NO_3^- + 0.29\times\mathrm{Temperature}$ ⋆	~1|habitat	190	0.16	0.35
4	Landscape	$\sqrt{r_a} = 0.30\times\log_{10}(\mathrm{Catchment})$	—	195	0.08	0.08
**5a**	**All**	$\boldsymbol{\sqrt{r_a} = 0.58\times\mathrm{Temperature} + 0.37\times NO_3^- - 0.29\times\mathrm{N} + 0.26\times\log_{10}(\mathrm{Catchment}) - 0.24\times\sqrt{\mathrm{DNA}}}$	**—**	**177 (192)**	**0.38**	**0.38**
5b	All	$\sqrt{r_a} = 0.51\times\mathrm{Temperature} + 0.47\times NO_3^- - 0.31\times\mathrm{N} - 0.27\times\sqrt{\mathrm{DNA}}$	—	179 (192)	0.35	0.35
5c	All	$\sqrt{r_a} = 0.10 + 0.93\times NO_3^- + 0.71\times\mathrm{Temperature} - 0.27\times\sqrt{nirS}$	~1+NO_3^-|lake	193	0.44	0.54
5d	All	$\sqrt{r_a} = 0.09 + 0.96\times NO_3^- + 0.63\times\mathrm{Temperature} - 0.25\times\sqrt{\mathrm{DNA}}$	~1+NO_3^-|lake	193	0.43	0.53
5e	All (+ habitats)	$\sqrt{r_a} = -0.12 + 0.80\times\mathrm{Carex} + 0.78\times\mathrm{Rocky} + 0.56\times NO_3^- + 0.43\times\mathrm{Temperature} - 0.27\times\sqrt{\mathrm{DNA}}$	—	179	0.37	0.37

Model	Explanatory var	Formula: Fixed part	Random part	AICc (gls)	Fixed R^2	Global R^2
		Potential denitrification rates (n = 52)				
lake		$\sqrt{r_p} = -0.13$	~1|lake	110	0	0.77
habitat		$\sqrt{r_p} = 0.24$	~1|habitat	152	0	0.20
6a	Sediment (molecular)	$\sqrt{r_p} = -0.52\times\log_{10}(nosZI) + 0.50\times\sqrt{nirS}$	—	139 (146)	0.26	0.26
6b	Sediment (molecular)	$\sqrt{r_p} = -0.06 + 0.30\times\sqrt{\text{nirS}} - 0.27\times\log_{10}(nosZI)$	~1|lake	111	0.08	0.79
6c	Sediment (molecular)	$\sqrt{r_p} = 0.42 + 0.68\times\sqrt{nirS} - 0.65\times\log_{10}(nosZI)$	~1|habitat	136	0.35	0.55
7a	Sediment (abiotic)	$\sqrt{r_p} = 0.49\times\text{C} + 0.43\times\delta^{15}\text{N}$	—	124 (132)	0.46	0.46
7b	Sediment (abiotic)	$\sqrt{r_p} = 0.48\times\text{OM} + 0.43\times\delta^{15}\text{N}$	—	125	0.45	0.45
7c	Sediment (abiotic)	$\sqrt{r_p} = 0.49\times\delta^{15}\text{N} + 0.48\times\text{N}$	—	125	0.44	0.44
8	Water (abiotic)	$\sqrt{r_p} = 0.48\times\text{Temperature} + 0.48\times\log_{10}(\text{SO}_4^{-2}) - 0.43\times\text{NO}_3^-$	—	115	0.53	0.53
9a	Landscape	$\sqrt{r_p} = -0.75\times\text{Altitude} - 0.65\times\log_{10}(\text{Catchment})$	—	117 (125)	0.52	0.52
9b	Landscape	$\sqrt{r_p} = -0.03 - 0.81\times\text{Altitude} - 0.63\times\log_{10}(\text{Catchment})\star$	~1|lake	110	0.44	0.77
**10a**	**All**	$\boldsymbol{\sqrt{r_p} = -0.77\times\log_{10}(nosZI) + 0.69\times\text{Temperature} + 0.64\times\sqrt{nirS} - 0.47\times\text{N} + 0.34\times\log_{10}(\text{SO}_4^{-2}) - 0.33\times\text{Altitude}}$	**—**	**90 (112)**	**0.77**	**0.77**
10b	All (+ habitats)	$\sqrt{r_p} = -0.14 - 0.77\times\log_{10}(\text{nosZI}) + 0.77\times\text{Carex} + 0.68\times\text{Isoetes} + 0.68\times\text{Temperature} + 0.66\times\sqrt{nirS} - 0.54\times\text{OM} + 0.36\times\log_{10}(\text{SO}_4^{-2}) - 0.30\times\text{Altitude}$	—	90 (114)	0.79	0.79
10c	All	$\sqrt{r_p} = 0.08 + 0.52\times\text{Temperature} - 0.50\times\text{Altitude} - 0.46\times\log_{10}(nosZI) + 0.41\times\sqrt{nirS}$	~1|lake	106	0.61	0.88
11	All (but molecular)	$\sqrt{r_p} = -0.99\times\log_{10}(\text{Catchment}) - 0.77\times\text{Altitude} + 0.51\times\log_{10}(\text{SO}_4^{-2})$	—	100	0.65	0.65
12	All (but landscape)	$\sqrt{r_p} = -0.73\times\log_{10}(nosZI) + 0.72\times\text{Temperature} + 0.57\times\sqrt{nirS} + 0.45\times\log_{10}(\text{SO}_4^{-2}) - 0.33\times\text{N}$	—	103	0.69	0.69

Note: Models with all components significant and with a ΔAICc value ≤ 2 for each set of explanatory variables are shown. Models with lake/habitat effect in the random part are shown when are similar or improve the fixed models. The intercept of all models was always not significant (p>0.05); they were not shown when having negligible values ($< 1 \times 10^{-15}$). In bold are indicated the best models, i.e. trade-off between being simple and explicative (AICc and ANOVA test). The star "★" indicate model components with p-value higher than 0.05, occurring in models 3e (p=0.058) and 9b (p=0.051).
